# Characteristics of *Mycoplasma pneumoniae* Pneumonia in Romanian Children

**DOI:** 10.3390/microorganisms13040883

**Published:** 2025-04-11

**Authors:** Alexandru Ioan Ulmeanu, Georgiana-Eugenia Ciuparu, Elena Roxana Matran

**Affiliations:** 1 Department of Pediatrics, “Carol Davila” University of Medicine and Pharmacy, 020021 Bucharest, Romania; alexandru.ulmeanu@umfcd.ro; 2 “Grigore Alexandrescu” Emergency Hospital for Children, 020021 Bucharest, Romania; georgiana-eugenia.ciuparu@rez.umfcd.ro

**Keywords:** *Mycoplasma pneumoniae*, community-acquired pneumonia, reactive infectious mucocutaneous eruption

## Abstract

Background/Objectives: *Mycoplasma pneumoniae* (*M. pneumoniae*), traditionally associated with mild community-acquired pneumonia in school-aged children, has experienced a delayed resurgence following the COVID-19 pandemic. The epidemiological and clinical characteristics of *M. pneumoniae* pneumonia in children within the context of this global resurgence have not been well established in Romania. Materials and Methods: This retrospective, single-center study analyzed children diagnosed with *M. pneumoniae* pneumonia who were hospitalized in the pulmonology department of “Grigore Alexandrescu” Emergency Hospital for Children in Bucharest from March to December 2024. Clinical, laboratory, and radiographic data were extracted from hospital records. *M. pneumoniae* infection was confirmed through polymerase chain reaction (PCR) multiplex panel detection or specific IgM antibody levels ≥ 10 AU/mL. Results: The final analysis included 63 patients who met the inclusion criteria. The cohort’s median age [IQR] was 12.6 [8–15] years, with 11.1% (n = 7) under 6 years old. The radiographic findings revealed a predominance of right lung involvement (52.4%, n = 33, *p* = 0.03) and a significantly higher prevalence of alveolar infiltrates compared to interstitial patterns (88.9%, n = 56, *p* < 0.001). Antibiotic choice did not significantly affect hospitalization duration. Pleural effusion emerged as a common complication, occurring in 27% (n = 17) of patients and associated with elevated admission leukocyte counts (*p* = 0.007). Rare extrapulmonary manifestations included meningoencephalitis (1.6%, n = 1) and reactive infectious mucocutaneous eruption (3.2%, n = 2). Notably, co-infections with other respiratory pathogens did not extend hospital stays. Conclusions: This study contributes to the evolving global epidemiological profile of *M. pneumoniae* infections in the post-pandemic era. It establishes a foundation for future multi-center analyses aimed at monitoring the changing epidemiology and clinical presentations of *M. pneumoniae* infections in pediatric populations.

## 1. Introduction

*Mycoplasma pneumoniae* (*M. pneumoniae*) is a common etiology of community-acquired pneumonia in school-aged children, with periodic epidemic surges occurring every few years [1].

Prior to the COVID-19 pandemic, *M. pneumoniae* was a prevalent cause of respiratory tract infections, with a global incidence of 8.61% between 2017 and 2020, as determined by direct diagnostic methods. A reduced incidence to 1.69% was observed between 2020 and 2021 due to the implementation of non-pharmaceutical interventions (NPIs) to combat COVID-19. Furthermore, even after the relaxation or discontinuation of NPIs, *M. pneumoniae* transmission continued to show long-term reduction, with an incidence of 0.70% between 2021 and 2022 and 0.82% between 2022 and 2023. Conversely, infections with other pathogens like the respiratory syncytial virus (RSV) experienced a resurgence, indicating an increase in community transmission [2].

The dynamic of *M. pneumoniae* infections showed an increased pneumonia outbreak across multiple geographic locations in late 2023 according to a global prospective surveillance group [the European Society of Clinical Microbiology and Infectious Diseases Study Group for Mycoplasma and Chlamydia Infections (ESGMAC), ESGMAC Mycoplasma pneumoniae Surveillance (MAPS) Study]. This late re-emergence is hypothesized to be the consequence of *M. pneumoniae* characteristics like slow growth, prolonged incubation period (2–3 weeks), and low transmission rates [3].

The Centre for Diseases Control and Prevention (CDC) reported in October 2024 that infections caused by *M. pneumoniae* have been on the rise since spring 2024, remaining elevated over the subsequent six months, with a peak incidence observed in August 2024. Notably, the infection rates were higher among children, increasing from 3.5% to 7.4% in those aged 5–17 years. The most remarkable increase, however, was seen in children aged 2–4 years, where rates rose from 1.0% to 7.2%. This finding is particularly significant, as *M. pneumoniae* has not historically been recognized as a major cause of pneumonia in this younger age group [4].

*M. pneumoniae* typically causes self-limiting respiratory infections and is commonly referred to as “walking pneumonia” [5]. However, in certain cases, the condition may progress to refractory *M. pneumoniae* pneumonia (RMPP), a more severe form of the disease. While a consensus definition for RMPP is lacking, it is generally characterized by the persistence or exacerbation of clinical symptoms, a prolonged fever, the deterioration of radiological findings, and the development of extrapulmonary complications. These manifestations occur despite appropriate macrolide antibiotic therapy for at least 7 days; however, markers like C-reactive protein (CRP), lactate dehydrogenase (LDH), erythrocyte sedimentation rate (ESR), neutrophils (%), and lymphocytes (%) may have predictive value [6].

Mild *M. pneumoniae* infections are common among children above 5 years of age. Respiratory infections with *M. pneumoniae* frequently manifest with an array of systemic and respiratory symptoms. The systemic manifestations typically include headache, fever, and muscle pain. Respiratory symptoms often encompass pharyngitis, non-productive cough, and mucopurulent expectoration. Young children and infants may exhibit wheezing and dyspnea in a small proportion. The extrapulmonary manifestations can involve the musculoskeletal, neurological, dermatological, cardiovascular, genitourinary, and hematological systems. Disease duration can vary between 7 and 10 days, and the prognosis is favorable for most patients [7,8].

The radiological features of *M. pneumoniae* are non-specific and consist of patchy areas of consolidation, interstitial infiltrates, or both [9]. Pleural effusion (PE) is a recognized complication of *M. pneumoniae* pneumonia, occurring with variable frequency and often associated with heightened inflammatory responses and prolonged febrile states. Recent research has proposed a dichotomous classification of PE based on pathogen genome isolation and cytokine profiles. This categorization distinguishes between PE that is genome-positive with elevated cytokine levels and PE that is genome-negative with lower cytokine concentrations [10]. Atelectasis was recently described in 40 out of 572 patients with *M. pneumoniae* and in a cohort of 122 children hospitalized in the Children’s Hospital of Xiamen between December 2015 and December 2018 [11].

The diagnosis of *M. pneumoniae* is achieved either by PCR testing, which represents the “gold standard”, with high specificity, sensitivity (detection of <100 colony-forming units (CFU)/mL), and rapid result acquisition; the presence of this pathogen in the upper respiratory tract may not necessarily indicate respiratory disease, as it can be found in 3% to 56% of asymptomatic children. Given the long incubation period and special media required, culture-based identification is not routinely used in practice. Rapid antigen testing identifies the pathogen with a cutoff limit of 1 × 10^3^ CFU/mL and has a lower sensitivity than PCR, although it is more efficient [12]. Serological diagnosis consists of specific antibody detection of either immunoglobulin (Ig) A, IgG, or IgM. The “gold standard” necessitates the collection of a serum sample after ≥2 weeks to observe the seroconversion or rise in antibody titer ≥ 4 times. IgM antibodies are usually detected as early as one week after the clinical onset, with a maximum increase in the third week; IgA antibodies increase, reach the maximum, and decline faster than IgM antibodies, and IgG antibodies commonly appear 2 weeks after the infection and are detectable for a long period [7,13,14].

In December 2023, the National Institute of Public Health issued a notification regarding the rising number of *M. pneumoniae* infections in Europe and the European Economic Area [15]. To our knowledge, this is the first study in Romania to systematically characterize pediatric *M. pneumoniae* respiratory infections in the post-COVID-19 era. The previous studies in Romania focused primarily on adult populations or pre-pandemic cohorts, resulting in limited data derived from pediatric populations and creating a gap in understanding the evolving epidemiology and clinical features of *M. pneumoniae* in pediatric patients. The aim of this study is to comprehensively evaluate the clinical, radiological, and laboratory characteristics of *M. pneumoniae* infection in pediatric patients, in the context of its re-surgency and elevated global incidence rates observed in the post-pandemic era. This research seeks to delineate regionally specific patterns of *M. pneumoniae* infection in Romania, contributing nationally representative data to the global epidemiological landscape. Furthermore, it will provide critical insights into the Romanian epidemiological context, enhancing international understanding of *M. pneumoniae* dynamics in the context of evolving post-COVID-19 respiratory pathogens transmission patterns.

## 2. Materials and Methods

### 2.1. Study Type and Area

This is a retrospective, single-center study conducted in Bucharest, the capital of Romania, located in the southeastern part of the country. Bucharest serves as the largest urban and medical hub in Romania, with a population over 2 million. The study period spanned form March to December 2024, capturing the post-pandemic resurgence of *M. pneumoniae* infections.

### 2.2. Study Population and Design

Pediatric patients diagnosed with laboratory-confirmed *M. pneumoniae* respiratory tract infection who were admitted to our pulmonology department were included. “Grigore Alexandrescu” Emergency Hospital for Children is the largest tertiary pediatric hospital in Bucharest. This institution serves as a regional referral center for severe pediatric cases, from both urban and rural environments. All the clinical, laboratory, and radiological data were extracted from the institutional electronic medical records system. Inclusion criteria comprised the confirmation of *M. pneumoniae* infection through either molecular diagnostic techniques (polymerase chain reaction, PCR assay of nasopharyngeal specimens from a multiplex panel including influenza virus, parainfluenzae virus 1, 2, 3 and 4, rhinovirus, respiratory syncytial virus, metapneumovirus, coronavirus, bocavirus, *Bordetella pertussis*, *Bordetella parapertussis*, *Chlamydia pneumoniae*, and *M. pneumoniae* through the Biofire^®^ method) or serological evidence (detection of pathogen-specific IgM antibodies through chemiluminescence immunoassay) defined as an IgM concentration ≥ 10 UA/mL in a patient with compatible clinical and paraclinical (biochemical and/or radiological) signs of respiratory infection. Subjects with inadequate or incomplete documentation were excluded from the final analysis.

Demographic parameters were recorded for all the participants. The clinical and laboratory variables collected included: interval from symptom onset to definitive diagnosis (measured in days); maximum recorded body temperature (expressed in degrees Celsius), peripheral blood leukocyte count (cells/mm^3^); serum CRP concentration (mg/dL); and serum procalcitonin level (ng/mL, available for 23 patients) at presentation.

The radiographic evaluation of pulmonary involvement was systematically categorized according to the anatomical distribution (bilateral, unilateral left, or unilateral right lung involvement) and predominant pathological pattern (presence or absence of alveolar or interstitial infiltrates). Additional variables documented included concurrent infection with other respiratory pathogens (viral, bacterial) detected through PCR panel testing. Identification of bacteria from the upper respiratory tract was based on cultures from the nasopharyngeal swabs. In addition, prior antimicrobial therapy before hospital admission, associated pulmonary complications including atelectasis, pleural effusion, and clinical manifestation of respiratory distress characterized by dyspnea were noted.

The in-hospital etiological treatment was standardized in the following three categories: azithromycin group, clarithromycin group, and newer quinolones (levofloxacin) group. Hospitalization length was also noted.

The study was approved by the Ethics Committee of our Institution (reference number #87/17.12.2024).

### 2.3. Statistics

Data were analyzed using SPSS v.26 (Chicago, IL, USA). Descriptive statistics were computed for both continuous and categorical variables. Continuous variables were evaluated for normality using the Kolmogorov–Smirnov test, given the sample size. Regarding the distribution for continuous variables, the mean/median with standard deviation (SD) or interquartile range (IQR) were used as appropriate. To analyze the association between categorical variables, we used the Chi-squared test. A Chi-squared goodness of fit test was performed to evaluate the distribution of categorical outcomes in our sample. Pearson’s correlation was used to evaluate the associations between the continuous variables that demonstrated a normal distribution. To compare the means and medians of the continuous variables between more than two groups, one-way ANOVA was used for the normally distributed data, and the Kruskal–Wallis test was employed for the non-normally distributed data. Independent-*T*-test was used to compare the means of the normally distributed variables between the two groups. Post hoc analysis was performed for ANOVA and the Kruskal–Wallis test, when significant differences were found between the groups. A *p* value < 0.05 was considered as the threshold for statistical significance.

## 3. Results

A total of 66 patients were recruited initially. Three were excluded due to insufficient data. The final analysis comprised 63 patients, with 30 (47.6%) boys. Median age at inclusion [IQR] was 12.6 years [8,9,10,11,12,13,14,15]. Three patients (4.8%) were diagnosed through serological testing. The patients’ characteristics are shown in Table 1. Baseline demographics and clinical characteristics of the study cohort are presented in Table 1 to provide context for subsequent analysis.

**Figure 1:**
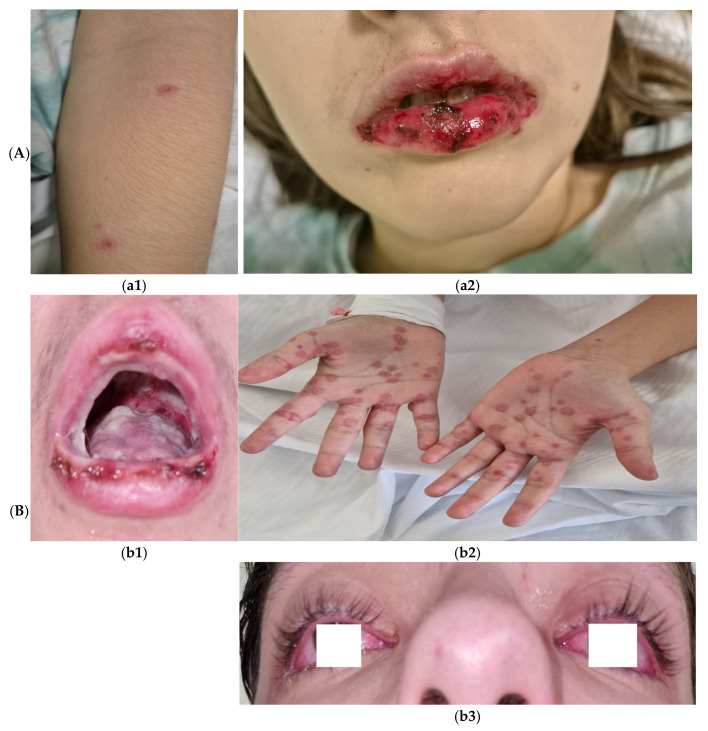
(**A**) Reactive infectious mucocutaneous eruption in a girl; (**a1**) targetoid skin lesions, (**a2**) oral mucositis. (**B**) Reactive infectious mucocutaneous eruption in a boy: (**b1**) oral mucositis, (**b2**) targetoid lesions, (**b3**) conjunctivitis.

**Figure 2:**
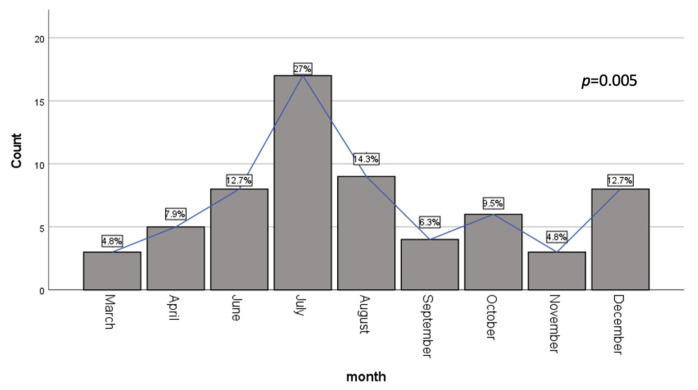
Monthly distribution of *Mycoplasma pneumoniae* respiratory infections.

**Figure 3:**
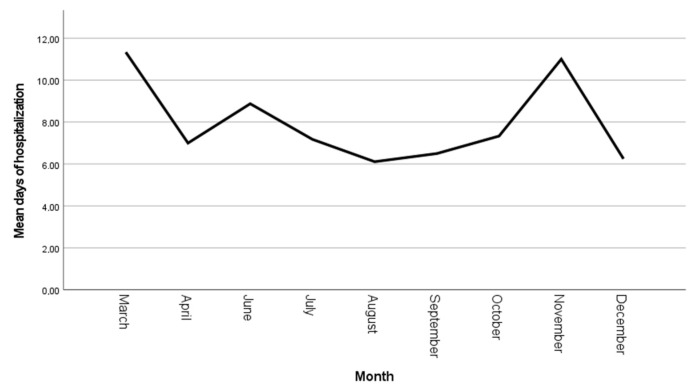
Monthly mean duration of hospitalization (days).

Complications included pleural effusion (PE) in almost a quarter of the patients (n = 17, 27%), meningoencephalitis in one patient (1.6%) and two cases (3.2%) of reactive infectious mucocutaneous eruption (RIME) syndrome (Figure 1). The monthly distribution of cases indicates a two-peaks prevalence, one in the summer (July–August) and one in the winter (December) (Figure 2) with a progressive reduction in the mean hospitalization duration from 11 ± 1.52 days in March 2024 to 6.2 ± 2.1 days in December (Figure 3). Identification of different pathogens detected on the PCR panel (viral/bacterial) or culture-based methods were observed in 19 (30.2%) of the patients, of which 8 (12.7%) had co-infections (pathogens detected concurrently with *M. pneumoniae* infection) with ≥1 virus and/or *Bordetella pertussis* (as identified on the multiplex PCR panel) while upper respiratory colonization (interpret as non-invasive microbial presence) was observed in 13 (20.6%) of patients (Table 2).

Duration of hospitalization was positively correlated with the fever value on admission (Pearson’s r coefficient = −0.247, *p* = 0.05) but not with the number of leukocytes (*p* = 0.181), CRP level (*p* = 0.178), or procalcitonin level (*p* = 0.698) or age (*p* = 0.107).

Regarding the duration of hospitalization in relation to the anatomical distribution of pulmonary involvement, right lung localization was associated with the longest hospitalization period compared to bilateral involvement, with means of 8.2 ± 2.7 vs. 5.7 ± 2.5 days (*p* = 0.014), but not significantly different when compared to left lung involvement, 7.2 ± 3.3 days, *p* = 0.201; there were no significantly statistic differences in the length of hospitalization between the left lung pneumonia group and bilateral involvement group (*p* = 0.182). Hospitalization length varied across the seasons, with the longest mean stay observed during the initial trimester, March–June, (8.7 ± 4.1 days). This reached the shortest duration in the middle trimester to 6.8 ± 2.1 days. The third trimester (October–December) had a mean hospitalization time of 7.4 ± 2.5 days. While ANOVA revealed no statistically significant differences across all the trimesters (*p* = 0.091), post hoc analysis identified a significant reduction in hospitalization time between the first and second trimesters (*p* = 0.029)

**Figure 4:**
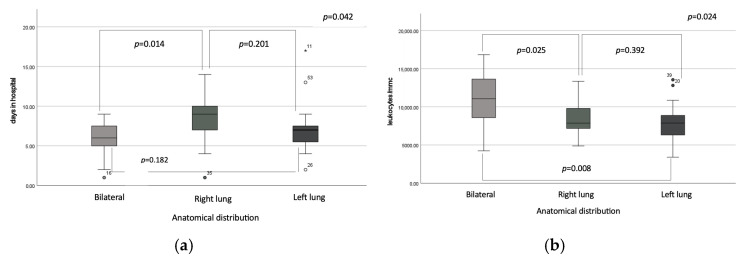
Box plot representation of: (**a**) duration of hospitalization and (**b**) leukocyte count distribution according to anatomical distribution of pneumonia. Circles and asterisks are outliers in the statistical analysis, circles are mild outliers, and asterisks are extreme outliers. The same explanation applies to the figure below.

**Figure 5:**
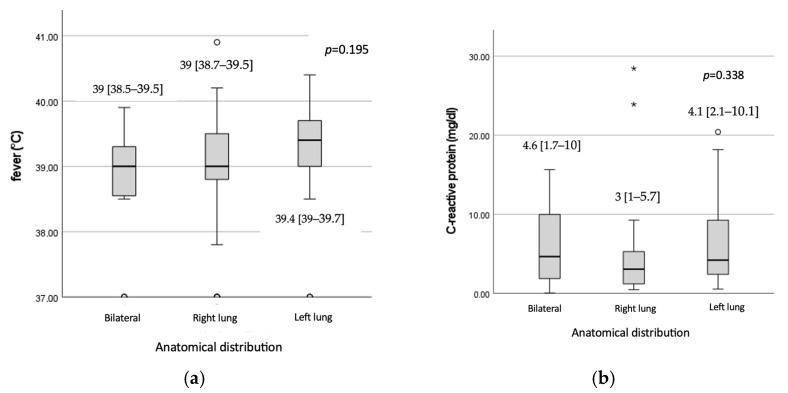

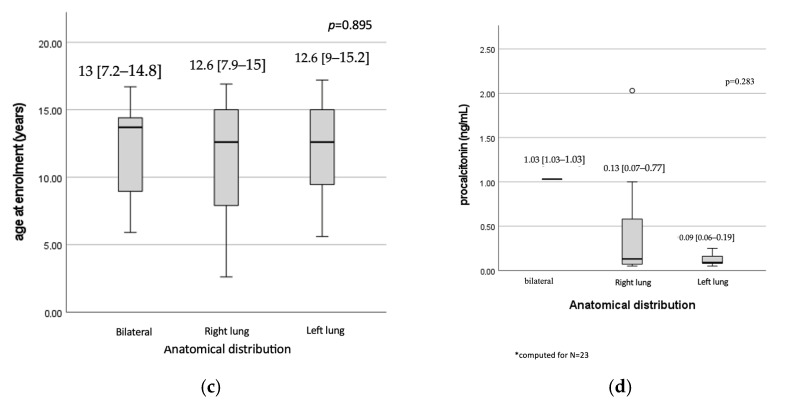
Box plot representation of: (**a**) fever values, (**b**) C-reactive protein, (**c**) age at enrolment, and (**d**) procalcitonin distribution according to anatomical distribution of pneumonia.

Conversely, leukocyte counts were significantly elevated in patients with bilateral pulmonary involvement (10,721 ± 3954.2 cells/mm^3^) compared to those observed in patients with right pulmonary involvement (8582.4 ± 2203 cells/mm^3^), *p* = 0.025 and those with left pulmonary involvement (7917.9 ± 2555 cells/mm^3^), *p* = 0.008, while we did not observe any significantly differences between left and right pneumonia group, *p* = 0.392 (Figure 4). No statistically significant differences were observed regarding fever values, 39 [38.5–39.6], *p* = 0.195), CRP levels (*p* = 0.338), procalcitonin levels (*p* = 0.283) or patients’ age (*p* = 0.895) in relation to the anatomical distribution of lesions (Figure 5).

When comparing Mycoplasma infection alone and co-infection with the viral and/or bacterial pathogens identified on the PCR multiplex panel, no significantly different distributions were observed regarding mean hospitalization time (7.3 ± 2.7 vs. 8.4 ± 4.1, *p* = 0.349), fever (39 [38.9–39.6] vs. 39.2 [37.2–39.6], *p* = 0.494), or biochemical parameters, including leukocyte count (8727.3 ± 2854.1 vs. 8950 ± 2557.4, *p* = 0.835), CRP (3.7 [1.3–8.5] vs. 4.2 [1–5.8], *p* = 0.536), and procalcitonin (0.13 [0.07–0.4] vs. 0.31) (only two patients had a procalcitonin level available) *p* = 0.791.

Etiological treatment with either azithromycin (n = 16), clarithromycin (n = 37) or newer quinolones (n = 8) did not influence the mean hospitalization length (7.1 ± 2.4 vs. 7.4 ± 2.9, respectively, 8.1 ± 2.7, *p* = 0.734). Two patients were excluded from this analysis because they received specific treatment after discharge.

Seven (11.1%) patients were ≤6 years of age. Leucocyte count was significantly higher among the children aged 6 years and younger as compared to the older children group (9790 [8680–12,930] vs. 7945 [6952.5–10,497.5], *p* = 0.046). There were no differences between the younger than 6 years of age group and >6 years group in terms of days of hospitalization (5.7 ± 2.1 vs. 7.6 ± 2.9, *p* = 0.09), fever (39 [37.8–39.7] vs. 39 [38.8–39.5], *p* = 0.645), CRP 1.1 [1.3–4.6] vs. 3.9 [1.2–8.9], *p* = 0.420), procalcitonin level (0.07 [0.07–0.07] vs. 0.13 [0.07–0.52], *p* = 0.522).

Most of the patients (n = 48, 76.2%) were hospitalized > 5 days. Hospitalization length (≤5 days or >5 days) could not be predicted by a cutoff value for leukocytes (AUC = 0.457), CRP (AUC = 0.567), procalcitonin (AUC = 0.580), or fever (AUC = 0.589) on admission as observed after ROC analysis.

Two patients (3.2%) were transferred into the Intensive Care Unit (ICU), one with respiratory failure and one with meningoencephalitis.

The occurrence of pleural effusion was associated with higher leukocytes count on admission (9322.8 ± 2846.6 vs. 7220.5 ± 2038.6, *p* = 0.007) but not with the fever grade (39.5 [38.9–39.8] vs. 38.9 [38.5–39.5], *p* = 0.152), CRP (3.1 [1.6–7.2] vs. 3.9 [1.2–8.6], *p* = 0.938), procalcitonin levels (0.1 [ 0.07–0.1] vs. 0.1 [0.07–0.85], *p* = 0.769), or age at inclusion (13.1 [8.3–15.4] vs.11.8 [7.9–14.8], *p* = 0.481). We did not find significant associations between localization of pneumonia and PE occurrence, *p* = 0.337.

## 4. Discussion

Due to the advent of highly sensitive diagnostic techniques enabling the real-time and precise detection of *M. pneumoniae*, coupled with increased recognition of this pathogen as a potential etiological agent in pediatric respiratory infections, the reported prevalence of *M. pneumoniae* infections has demonstrated a progressive increase over the past decade. Our findings indicate that *M. pneumoniae* infection is prevalent among school-aged children, aligning with the previous studies that have reported a higher incidence in children older than 5 years. For instance, Kutty et al. demonstrated a significant prevalence of *M. pneumoniae* among children hospitalized with community-acquired pneumonia, particularly in those aged 5–17 years [16], similar to the reports of Shah et al. [17]. Conversely, Zhang et al. reported a higher incidence (41.3%) in children aged 5 years and younger, which differs from the observations in our study [1]. This discrepancy may be attributed to the elevated co-circulation of influenza, RSV and *M. pneumoniae* infections during the peak of the epidemic in China in the fall of 2023. Such regional differences suggest the importance of considering local epidemiological factors when interpreting variations in *M. pneumoniae* incidence across age groups.

Our study revealed a lower rate of viral co-infection compared to previous reports in the literature. For instance, Chen et al. observed co-infection in 14.5% of hospitalized children with *M. pneumoniae* respiratory tract infections, with RSV, bocavirus, and parainfluenzae being the most prevalent [18]. Notably, our findings corroborate the seasonal pattern reported by Chen et al., with peak incidence occurring during the summer months, although different etiologies were observed. More recent studies have reported varying co-infection rates, with some indicating rates as high as 38.75% [19]. The lower co-infection rate observed in our study may be attributed to several factors, including regional variations in pathogen prevalence or temporal changes in infection dynamics.

The mean time of hospitalization observed in our study aligns with the findings of Wang et al. They found different periods, based on disease severity, between 6.54 ± 2.58 days for mild cases and 9.25 ± 2.53 days for severe *M. pneumoniae* pneumonia [20]. A progressive reduction in mean hospitalization time was observed during the third trimester (October–December), likely reflecting the increasing clinician familiarity with disease progression patterns. However, a notable exception occurred in November, marked by a temporary increase in hospitalization length attributable to two cases of RIME syndrome. This isolated deviation highlights the impact of rare, severe extrapulmonary manifestations on variations in hospitalization measure, and it underscores the importance of accounting for atypical presentations when analyzing trends in infectious disease management.

Consistent with the previous findings, the mean leukocyte count at enrolment was within the normal range. However, our results are different from those reported by Youn et al., as we observed that children over six years of age presented with higher leukocyte counts compared to younger children. This discrepancy may be attributed to the fact that a substantial proportion of our study cohort received antibiotic treatment prior to enrolment, potentially influencing the observed leukocyte dynamics. The higher leukocyte counts in older children may reflect age-specific variations in immune responses or differences in disease severity. However, the confounding effect of pre-enrolment antibiotic use must be considered when interpreting these results [21].

Median CRP levels at baseline were moderately elevated, approximately seven times the normal value, consistent with the previous studies linking increased CRP levels to pneumonia severity [22]. However, contrary to the earlier findings, our analysis did not reveal a significant relationship between the severity of pneumonia, as assessed by its anatomical distribution, and the degree of inflammation. This observation suggests that while CRP is a reliable marker of systemic inflammation, its utility in predicting localized pulmonary involvement may be limited.

Contrary to the findings reported by Chen et al. [18], our study did not reveal statistically significant differences in the inflammatory markers (CRP levels and leukocyte counts) or length of hospitalization between the patients with isolated *M. pneumoniae* infections and those with co-infections involving other pathogens. We speculate that this can be explained by the fact that the association of additional pathogens does not substantially alter the inflammatory response or clinical course compared to only *M. pneumoniae* infections.

Kutty et al. observed no statistically significant differences in the length of hospitalization between children who received antibiotics against *M. pneumoniae* and those who did not [16]. Our study demonstrated that the choice of specific antimicrobial therapy did not significantly impact the duration of hospitalization. These observations across studies suggest that factors beyond antibiotic selection may play a more crucial role in determining the length of hospital stay for pediatric patients with *M. pneumoniae* pneumonia. Several potential explanations can be proposed, including the host immune response, disease severity, timing of antibiotic introduction, as well as factors like on-demand discharge or the occurrence of complications. Thus, the variability in hospitalization length may be influenced by factors beyond the direct pathophysiological impact of the infection, potentially confounding its utility as a standalone measure for disease course and severity assessment. We noticed an isolated increase in mean duration of hospitalization in November (11 ± 2 days); the most probable explanation for this observation is that, in this specific time frame, two cases of RIME syndrome were identified and were associated with a prolonged hospitalization time.

The radiological manifestations of *M. pneumoniae* pneumonia are predominantly categorized as bronchopneumonia or segmental/lobar pneumonia/atelectasis, reflecting distinct pathological processes. As demonstrated by Huang et al., bronchopneumonia represents the most frequent imaging pattern (59.6% of cases), while consolidation/atelectasis correlates with severe clinical outcomes, including prolonged hospitalization, elevated inflammatory markers (e.g., leukocytosis, CRP), and complications such as necrotizing pneumonia or bronchiolitis obliterans [23]. Contrary to the reported bilateral involvement being the most prevalent [24], the right lung predominance observed in our cohort is more likely due to anatomical factors (shorter right bronchus) facilitating pathogen deposition. Bilateral involvement indicates diffuse disease progression, correlating with higher leukocyte counts as observed in our research but not prolonged hospitalization, as reported by Huang et al. [23]. Variability in radiological classification may account for interstudy discrepancies. However, it is suggested that the severity of *M. pneumoniae* pneumonia is intrinsically tied to its radiological phenotype, with consolidation/atelectasis and bilateral involvement serving as markers of systemic inflammation and clinical severity. A unified imaging-based classification system, as proposed by Huang et al., could improve prognostic stratification and management strategies [23].

Pleural effusion represents a significant complication of *M. pneumoniae* pneumonia, serving as a marker of disease severity and prolonged clinical course. Its reported prevalence varies across studies, ranging from 4 to 28% in adults [25] and 20.3 to 20.7% in children in studies with a small sample size [26]. Shen et al. reported a proportion of 19% of children with *M. pneumoniae*-related parapneumonic pleural effusion, out of a total of 59 children with bacterial pneumonia complicated by PE and empyema [27]. Lee et al. observed that 65% of ICU-admitted patients presented with pleural effusion, compared to only 10% of non-ICU patients [28]. Lin et al. reported that 6.7% of patients with pleural effusion or empyema were *M. pneumoniae* PCR-positive [29]. In our research, PE did not require ICU admission. Contrary to the previous findings [26], we did not observe any differences in the CRP markers or in the hospitalization time between groups with and without PE, although leukocyte count was higher among the PE group. Differences in observations may be explained by various diagnostic modalities [X-ray or computed tomography (CT)], antibiotic regimens and the time of their initiation, age at enrolment, or regional pathogen dynamics. While pleural effusion remains a critical indicator of *M. pneumoniae* pneumonia severity, its clinical significance must be interpreted within the context of regional epidemiology, diagnostic practices, and treatment protocols. 

Central nervous system (CNS) complications associated with *M. pneumoniae* are more prevalent in pediatric populations and have been linked to elevated morbidity and mortality. Pathogenesis primarily involves immune-mediated mechanisms, particularly molecular mimicry, where antibodies targeting *M. pneumoniae* cross-react with host neuronal components such as myelin glycolipids, leading to autoimmune-driven neurological damage [30]. The direct invasion of the parenchyma, vascular injury, and a hypercoagulable state have also been noted [31,32]. Neurological involvement has been reported in 1–10% of hospitalized patients. *M. pneumoniae* is a common cause of encephalitis in children and is reported in 5–10% of cases. Younger children, especially those under 10 years of age are most often affected. Encephalitis presents in the following two distinct patterns: early-onset, characterized by acute neurological symptoms concurrent with respiratory infection, and late-onset, which manifests days to weeks after the initial symptoms and is often immune-mediated [31].

Similar to its respiratory manifestations, *M. pneumoniae* encephalitis often occurs in an epidemic pattern [33].

Our patient developed meningoencephalitis on the fifth day of hospitalization (day 11 of illness), manifesting with fever, diplopia, photophobia, strabismus, nuchal rigidity, and vomiting. A CT of the brain revealed no structural abnormalities. Cerebrospinal fluid (CSF) analysis via lumbar puncture demonstrated a positive Pandy reaction. The patient was transferred to the ICU for five days, where she received broad-spectrum antibiotics and intravenous corticosteroids, resulting in favorable clinical improvement.

RIME syndrome was first defined as a distinct clinical entity in 2015; historically, it was described as Stevens–Johnson syndrome and toxic epidermal necrolysis. Pathogenesis involves molecular mimicry between *M. pneumoniae* P1 adhesin and keratinocyte antigens, immune complex deposition, B-cell activation, genetic susceptibility, and triggers from infections (e.g., *M. pneumoniae*, *Chlamydia pneumoniae*, viruses) or medications. Clinically, RIME preferentially affects mucous membranes, with oral (94–100%), ocular (82–92%), and urogenital (63–78%) involvement, while cutaneous manifestations are variable—absent in 34% of cases or polymorphic (e.g., targetoid, vesiculobullous) in 47% [34]. In a meta-analysis by Canavan et al., the mean age at diagnosis was 11.9 ± 8.8 years with a male predominance [35]. In our study, the median age was similar at 12 [9.0–15] years, along with an equal distribution between the genders, likely due to the reduced incidence in our study group. One patient presented with mucositis, ocular involvement, and uro-genital lessions. RIME syndrome exhibits a seasonal pattern, with a predominance of cases reported during winter months. This seasonal distribution is associated with extended hospitalization periods [36]. Our observations align with this trend, as we documented cases in late November and were characterized by prolonged hospital stays, with a mean of 7 ± 2.4 days.

Despite its significant contribution to the current literature, the present study is subject to certain limitations. Primarily, its retrospective design may introduce selection biases in data collection, including incomplete data and inconsistences in treatment strategies. The absence of a control group limits the possibility to generalize results beyond the study group population. These methodological limitations should be considered when interpreting the findings and their broader applicability to clinical practice. Additionally, hospitalization duration may have been influenced, in some cases, by patient-requested discharge or, conversely, by prolonged stays due to extrapulmonary complications. A significant limitation was the complete unavailability of testing kits for *M. pneumoniae* during May 2024. This methodological constraint may have impacted on the ability to accurately perform a diagnosis; thus, including cases from this period potentially led to an underestimation of the true incidence of *M. pneumoniae* infections in the study population. Furthermore, the study did not include cases of COVID-19 co-infections that may have influenced the clinical severity of *M. pneumoniae* pneumonia, according to the post-pandemic framework. Future research should evaluate the interactions between these pathogens in regions where they co-circulate. We also highlight a specific limitation related to the study period regarding co-infections with winter-predominant pathogens such as RSV or influenza. Future studies should include data collected over a full calendar year.

These limitations underscore the need for cautious interpretation of the findings and place emphasis on the areas for improvement in future prospective studies on *M. pneumoniae* infection.

## 5. Conclusions

*M. pneumoniae* infections have re-emerged as a significant public health concern following the relaxation of pandemic restrictions, even though their resurgence has lagged behind other respiratory pathogens. Notably, the demographic profile of affected individuals has shifted toward younger age groups, with children under 10 years of age becoming increasingly susceptible, although these cases often present with reduced systemic inflammatory markers. PE, while historically considered rare, has emerged as a common complication. Prolonged hospitalization has been associated with right lung involvement, which may be influenced by the frequency of this distribution. Leukocyte counts upon admission appear to be correlated with both the anatomical distribution patterns and PE development, suggesting that hematological markers may serve as early indicators of disease severity. Co-infections with bacterial or viral pathogens did not exacerbate clinical severity or amplify inflammatory responses, a finding consistent with the recent studies highlighting the independent pathogenic role of *M. pneumoniae* in driving outcomes.

This study contributes to the evolving global epidemiological understanding of *M pneumoniae* infection. Our research adds data to the global map of *M. pneumoniae* infection epidemiology and establishes a foundation for future multi-center analyses to monitor changing epidemiology and clinical presentations of *M. pneumoniae* infection.

## Figures and Tables

**Table 1:** Baseline patients’ characteristics.

	Study Cohort (n = 63)	*p*-Value
Sex [n (%)]		
Male	30 (47.6)	0.705
Female	33 (52.4)	
Residence [n (%)]		
Urban	50 (79.4)	<0.001
Rural	13 (20.6)	
Median age at inclusion, years [IQR]	12.6 [8–15]	-
Median body temperature, degrees Celsius [IQR]	39 [38.8–39.6]	-
Mean number of leukocytes (cells/mm^3^ ± SD)	8756 ± 2799.8	-
Median serum C-reactive protein level, mg/dL [IQR]	3.7 [1.3–8.4]	-
Median serum procalcitonin level *, ng/mL [IQR]	0.13 [0.07–0.5]	-
Disease duration before admission [days; median, IQR]	7 [5–9]	-
Mean number of hospitalization days (± SD)	7.5 ± 2.9	-
Febrile on admission [n (%)]		
Yes	60 (95.2)	<0.001
No	3 (4.8)	
Alveolar infiltrates		
Yes	56 (88.9)	<0.001
No	7 (11.1)	
Interstitial pattern		
Yes	7 (11.1)	<0.001
No	56 (88.9)	
Anatomical distribution [n (%)]		
Bilateral	11 (17.5)	0.03
Right lung	33 (52.4)	
Left lung	19 (30.1)	
Pleural effusion [n (%)]		
Yes	17 (27)	<0.001
No	46 (73)	
Atelectasis [n (%)]		
Yes	2 (3.2)	<0.001
No	61 (96.8)	
Previous antibiotic therapy [n (%)]		
Yes [18 (43.9%) amoxicillin or amoxicillin + clavulanate, 19 (46.3%) cephalosporins, 3 (7.3%) macrolides and 1 (2.4%) with TMT/SMX)	41 (65.1)	0.017
No	22 (34.9)	
Concurrent respiratory infection [n (%)]		
No	51 (81)	<0.001
Yes	12 (19)	
Respiratory distress [n (%)]		
No	51 (81)	<0.001
Yes	12 (19)	
Respiratory failure [n (%)]		
No	10 (15.9)	<0.001
Yes	53 (84.1)	

IQR, interquartile range; SD, standard deviation; TMT/SMX, trimetoprime/sulphametoxazole. * Available only for 23 (36.5%) patients.

**Table 2:** Co-infections and upper respiratory tract colonization in the study cohort.

Co-Infection	N, % *
Rhinovirus/Enterovirus	7 (11.1)
Parainfluenzae 3	2 (3.1)
RSV	1 (1.6)
*Bordetella pertussis*	1 (1.6)
**Upper respiratory tract colonization**	
*Acinetobacter baumanii*	1 (1.6)
MSSA	7 (11.1)
*Streptococcus pneumoniae*	1 (1.6)
*Haemophilus influenzae*	2 (3.2)
*Streptococcus pyogenes*	1 (1.6)
*Candida albicans*	1 (1.6)

MSSA, methicillin-sensitive *Staphylococcus aureus*; RSV, respiratory syncytial virus. * patients may be included in more than one category.

## Data Availability

The original contributions presented in this study are included in the article. Further inquiries can be directed to the corresponding author.

## Abbreviations

The following abbreviations are used in this manuscript:

CNS	Central nervous system
CRP	C-reactive protein
CSF	Cerebrospinal fluid
CT	Computed tomography
IQR	Interquartile range
ICU	Intensive Care Unit
*M. pneumoniae*	Mycoplasma pneumoniae
PE	Pleural effusion
PCR	Polymerase chain reaction
RIME	Reactive infectious mucocutaneous eruption
RSV	Respiratory syncytial virus
SD	Standard deviation
TMT/SMX	Trimetoprime/sulphametoxazole
